# Rehabilitation needs prior to adjuvant chemotherapy in a rural Swedish population

**DOI:** 10.2340/jrm.v57.43516

**Published:** 2025-09-03

**Authors:** Linn NORRGÅRD, Bertil AXELSSON

**Affiliations:** 1 Research and Development Unit, Östersund Hospital, Östersund; 2 Department of Interventions and Diagnostics, Umeå University, Umeå, Sweden

**Keywords:** cancer rehabilitation, caregivers, chemotherapy, quality of life, rehabilitation needs, rural population, well-being

## Abstract

**Objective:**

To assess well-being and unmet needs among participants, and examine differences between patients and their next of kin.

**Subjects:**

Cancer patients pre-adjuvant chemotherapy (*n* = 231) in rural northern Sweden, and their next of kin (*n* = 204).

**Methods:**

Well-being and unmet needs were assessed using the Edmonton Symptom Assessment System (ESAS), Functional Assessment of Cancer Therapy–General (FACT-G), Functional Assessment of Chronic Illness Therapy–Spiritual Well-Being Scale (FACIT-Sp-12), Hospital Anxiety and Depression Scale (HADS), and Cancer Survivors’ (Partners’) Unmet Needs measure (CaSUN)/(CaSPUN). Descriptive methods summarized sociodemographic, cancer-specific, and psychosocial data. Non-parametric statistical tests examined the differences between patients and next of kin.

**Results:**

The HADS indicated that one-quarter of patients and one-third of next of kin possibly/probably had anxiety, and one-seventh of participants possibly/probably had depression. One-third of participants experienced no/minimal impact of cancer on well-being. The CaSUN identified unmet needs in all domains, but mostly in information and psychosocial domains. At least 15% of patients/next of kin reported unmet needs in 12/26 items. For most items, next of kin reported unmet needs at a similar/higher frequency than patients (10–25%).

**Conclusion:**

These findings underscore the importance of assessing well-being and rehabilitative needs in patients and their next of kin before chemotherapy, to identify those who may benefit from early professional support.

## LAY ABSTRACT

Impairments from cancer and its treatments are common and can be addressed through rehabilitation. This study used a combination of questionnaires to assess different aspects of well-being and unmet rehabilitation needs. Participants were cancer patients about to start adjuvant chemotherapy, and their closest partner and/or next of kin, in northern rural Sweden. Results of the Hospital Anxiety and Depression Scale (HADS) identified 14–34% of participants with increased psychological distress. Concurrently 33% reported no/minimal impact on well-being, and that they felt well. Unmet needs were identified in all domains, but most frequently in the information and psychosocial domains. For the majority of items, next of kin reported unmet needs as frequently as or more often than the patients. Our results suggests that assessments of well-being and unmet needs should be prioritized for patients and their next of kin, before treatment, to identify those who may benefit from early professional support.

Every year, almost 75,000 individuals in Sweden are affected by a cancer diagnosis and subsequent treatment (1). Assessment of supportive care needs is an essential component of providing comprehensive care to these individuals. Supportive care needs can be defined as needs for help of the cancer patient and their next of kin (NoK) throughout the cancer trajectory. These needs differ between individuals and may present at time of diagnosis, during treatment, or even long after completion of treatment, and may change over time (2).

In Sweden, 9% of the population live in the northern region’s 4 counties covering 54% of Sweden’s total land area (3). Being part of a rural community may affect a person’s supportive care needs. It can provide closer relationships (4) and better functional and social/family quality of life (QoL) (5, 6), but can also contribute to worse physical QoL (5) and less access to supportive care (13). Significant travel distances to access cancer care, and lack of local supportive care, can affect treatment decisions and follow-up, and impact both the well-being and health of patients (6).

Some studies report no significant differences in psychosocial well-being (5, 6), needs, and QoL (6) between rural and urban cancer survivors. In general, most studies in rural cancer survivors have been conducted in Australia, the USA, and Canada, with a post-treatment focus. Few studies have included the rural cancer patients’ families or NoK (5, 6).

Despite the well-documented benefits of cancer rehabilitation on physical and psychological well-being, several studies have highlighted its underutilization in cancer care (7, 8). A recent qualitative study conducted in rural Iceland revealed that both the structured rehabilitation services and information concerning rehabilitation were often lacking. Yet patients expressed a need for these resources to regain strength and a sense of normality. Findings also suggests the importance of continuous monitoring of needs as rehabilitation must address complex and evolving challenges (8).

Optimal well-being and/or QoL are common goals in cancer rehabilitation, both being multidimensional and being assessed differently across disciplines (9–12). Well-being can include 2 main criteria: objective functioning (physical and cognitive capacity) and subjective values (i.e., the patient’s view of his/her own good, goals, and wishes). A hybrid approach combining these criteria has been suggested to benefit patients (13). In this study, we chose well-being over QoL, assuming it to be a more relatable concept for people in northern Sweden.

We initiated this study to gain a better understanding how cancer care affects the well-being and supportive needs of patients and NoK, to tailor a routine assessment and appropriate rehabilitative support interventions for cancer patients in rural northern Sweden. Primarily, we aimed to assess the well-being, and prevalence of unmet needs, of cancer patients and their NoK, pre-adjuvant CT. A secondary aim was to examine whether there were any differences in well-being and needs between patients and their NoK.

## METHODS

The Rehabilitation Needs of Patients Receiving Adjuvant Chemotherapy (REPAC) study was started in northern Sweden in 2015 (14). Cancer patients receiving postoperative adjuvant CT and their NoK were invited to participate in a longitudinal cohort study to assess well-being and rehabilitation needs alongside cancer treatment. Data were collected between 2015 and 2021 in Norrbotten and Jämtland, 2 comparable, large, and sparsely populated counties.

### Context

The definition of rural or remote areas varies in the literature, with different classifications depending on country and region (5). With a mean of 2.6 inhabitants per square kilometre, the 4 counties in northern Sweden can be regarded as predominantly rural (3). Cancer care in 2 of these counties, Norrbotten and Jämtland, is comparable. It is centralized to a hospital in the main city which lacks an oncology department, and the patients have thus been dependent on visiting oncology consultants from the University Hospital of Umeå (290–360 km away). The cancer rehabilitative support in these 2 counties has historically focused on patients who struggle to return to everyday life after completion of chemotherapy (CT), particularly patients with severe rehabilitative needs. Support has been provided based on individual needs but has mainly been offered in response to an active request on the part of the patient.

Östersund city (53,992 inhabitants) is the only city in Jämtland, while Norrbotten has 4 smaller towns (< 20,000 inhabitants) and Luleå (49,646 inhabitants) (15). Inhabitants are accustomed to travelling great distances; some patients live 3–4 hours’ one-way travel distance from the nearest hospital.

### Procedure and participants

All consecutive cancer patients who were recommended adjuvant or neo-adjuvant CT were initially approached by nurses working in the outpatient clinics. Inclusion criteria were epithelial cancer diagnosis; curative surgery completed but adjuvant CT not yet started, or about to start preoperative CT (in advance of surgery); minimum age 18 years; cognitively intact; and fluent in Swedish. Patients with signs of recurrence or medical conditions that contraindicated CT were excluded.

After being given information on the study, 231 patients (57% of those approached) gave informed written consent. All included patients were subsequently asked to invite a partner and/or NoK to participate. In this study, both partners and NoK will henceforth be referred to as NoK. Of 231 patients, 172 chose to invite 1 or more NoK; in total 204 NoK were included after informed written consent. Sociodemographic data (gender, age, county, civil status, education level, and occupation) and cancer-specific data (cancer type, CT intent, and type) were collected at baseline. Also collected at baseline were potential background factors for vulnerability, including previous anxiety or depression, previous alcohol or drug abuse, recent adversity or loss, and having/not having someone to share their thoughts with.

Participants were asked to complete the questionnaire package at baseline (before/at first CT treatment), and again 2–3 months into the treatment, and subsequently 1–2, 6, and 12 months after treatment. The questionnaires were delivered by mail for the participants to complete at home. The current study examines results from the first questionnaire package (Q1), distributed before the start of CT.

### Questionnaires/measures

The construction of the questionnaire package was guided by a literature review and clinical practice. The 5 questionnaires listed below were combined to address various aspects of well-being and rehabilitation needs.

1. ESAS: Edmonton Symptom Assessment System
2. HADS: Hospital Anxiety and Depression Scale
3. FACIT-SP-12: Functional Assessment of Chronic Illness Therapy–Spiritual Well-Being Scale
4. FACT-G: Functional Assessment of Cancer Therapy–General
5. CaSUN/CaSPUN: Cancer Survivors’ Unmet Needs measure/Cancer Survivors’ Partners’ Unmet Needs measure

*Edmonton symptom assessment system.* The ESAS is a thoroughly validated and commonly used tool for symptom screening and longitudinal monitoring of symptoms in clinical cancer settings (16). It includes items on 10 patient-rated (0–10) symptoms including anxiety and depression. To provide insight into both physical and psychological areas of well-being, all ESAS items were analysed. Cut-offs for no, mild/moderate, and severe impact were set to 0–2, 3–6, and 7–10.

*Hospital anxiety and depression scale.* The HADS is a 14-item questionnaire that measures overall psychological distress (range 0–42). The instrument includes a subscale on depression (HADS-D) and another on anxiety (HADS-A), with 7 items each (range 0–21). A summed score of 8–9 (for each subscale) was used as an indicator for a possible case of anxiety/depression, and ≥ 10 as indicating a probable case (17). In this study, both possible and probable anxiety/depression were considered to have an impact on psychological well-being.

*Functional assessment of chronic illness therapy–spiritual well-being scale (version 4).* Spiritual well-being has emerged as an important aspect of well-being. The FACIT-Sp-12 questionnaire is the most widely used measure of spiritual well-being among individuals with cancer (18). It comprises 12 questions rated on a 5-point Likert scale, from 0 = not at all, to 4 = very much. In this study 4 of 12 items were chosen to assess aspects of meaning and feeling at ease as essential parts of general well-being.

*The functional assessment of cancer therapy–general (version 4).* The FACT-G is a questionnaire consisting of 27 items on health-related quality of life (HRQoL) in cancer patients. The instrument’s reliability and validity have repeatedly been confirmed and the sensitivity for longitudinal use demonstrated. The questionnaire items are divided into 4 domains: physical well-being, social/family well-being, emotional well-being, and functional well-being (19, 20). Responses are rated on a 5-point Likert scale from 0 = not at all, to 4 = very much. We chose 7 of 27 items to assess aspects of physical, functional, and emotional well-being, complementary to those collected by other questionnaires.

*Cancer survivors’ unmet needs measure/cancer survivors’ partners’ unmet needs measure.* The CaSUN is a validated and widely used questionnaire that assesses cancer-related needs experienced in the preceding month. It comprises 35 items rated “no need/not applicable”, “met need”, or “unmet need”. If a need is unmet, it is further rated as weak, moderate, or strong (21). The items are grouped into 5 domains: psychological or existential survivorship (14 items), comprehensive care (6 items), information (3 items), QoL (2 items), and relationships (3 items). The remaining 7 items are not grouped into a domain but still contain useful clinical information (22). In this study, all comparable items (26 out of 35) in the CaSUN and CaSPUN were analysed (23, 24). Mean values for CaSUN/CaSPUN data were calculated, ranging from 1 = least important, to 3 = most important to visualize the strength of an unmet need.

To identify less affected participants, we created a summed score criterion for no/minimal impact on well-being: no impact on the ESAS items well-being and QoL (score 0–2), combined with a summed score < 8 for the HADS-A and HADS-D (= no probable case).

Missing data were not substituted, resulting in a small variation in the number of reporting participants per item in the results section.

### Statistical data analysis

Power calculations were two-sided and based on clinical relevance. To ensure 80% power, and detect a difference between 7% and 20% in compared groups, the study needed to include 108 participants per group.

Descriptive statistical analysis was performed to summarize sociodemographic, cancer-specific, and psychosocial data. To examine sociodemographic differences between patients and NoK we used Fisher’s exact test (2 sets of categorical data), a χ^2^ test (categorical data) and the Mann–Whitney *U* test (continuous data). For differences between patients and NoK in questionnaire scores, we used a χ^2^ test (ESAS, HADS, and CaSUN) and Mann–Whitney *U* test (FACT-G and FACIT-Sp-12). Non-parametric statistical tests were performed because the obtained data were ordinal and had a skewed distribution.

A *p*-value of < 0.05 was considered statistically significant. Statistical analysis was performed using StatView statistical software version 5.0.1 (SAS Institute, Cary, NC, USA).

## RESULTS

This study included 231 patients and 204 NoK. The characteristics of participants are described in Table I. The patients were on average older than the NoK. The majority of patients were women (78%); most had breast cancer (69%) and were married or cohabiting (71%). Among the NoK the gender distribution was more even (53% men and 47% women). Almost one-third of patients and one-fourth of NoK reported previous anxiety and/or depression. An analysis of eligible vs included patients was performed, and no significant differences were found regarding age, sex, diagnosis, or county.

**Table I:** Sociodemographic and disease-related characteristics of patients and next of kin (NoK) at baseline. Fisher’s exact test and χ^2^ test were used for categorical data. Mann-Whitney U test was used for continuous data (age).

Characteristic	Variable	Pats, *N* (%)	NoK, *N* (%)	*P*-value
Gender	Men	53 (22)	108 (53)	< 0.001
	Women	178 (78)	96 (47)	
Age, yrs, median (range)		63 (33-83)	56 (18-84)	< 0.0001
County	Jämtland	107 (46)	101 (50)	0.50
	Norrbotten	124 (54)	103 (50)	
Civil status	Married	119 (51)		
	Cohabiting	45 (20)		
	Living apart	12 (5)		
	Single	55 (24)		
Education level	Elementary school	61 (27)	46 (23)	0.207
	High school	69 (31)	72 (35)	
	College/university education	96 (42)	86 (42)	
Occupation	Student	0 (0)	6 (3)	0.018
	Working	121 (54)	118 (58)	
	Retired	105 (46)	71 (35)	
	On sick leave	2 (0)	2 (1)	
	Unemployed	1 (0)	5 (3)	
Cancer type	Breast	160 (69)		
	Colon	58 (25)		
	Other	13 (6)		
Treatment	Adjuvant	223 (97)		
	Neo-adjuvant	7 (3)		
Chemotherapy	(A)	50 (22)		
	(B)	84 (36)		
	(C)	21 (9)		
	(D)	7 (3)		
	(E)	69 (30)		
Previous A/D	Yes	70 (30)	52 (25)	0.22
Previous A/D medication	Yes	50 (22)	28 (14)	0.02
Previous alcohol/drug abuse	Yes	9 (4)	3 (1)	0.11
Recent adversity or loss	Yes	93 (40)	91 (45)	0.58
Having someone to share thoughts with	Yes	218 (94)	199 (98)	0.1

A/D = anxiety/depression; NoK = next of kin (*N* = 202-204); Pats = patients (*N* = 227-231)

Chemotherapy (A) = pyrimidine analogues: Capecitabine, Capecitabine-Oxaliplatin, FEC-75, FLOX, FOLFOX, Gemcitabine, Teysuno; (B) Docetaxel + Epirubicin-Cyclophosphamide; (C) Epirubicin-Cyclophosphamide; (D) = other: Carboplatin, Paklitaxel, Vinorelbine; (E) not specified.

### Well-being

The criterion for no/minimal impact on well-being was met by one-third of participants: 33% of patients and 36% of NoK. Concurrently, a moderate proportion of participants, both patients and NoK, reported significant psychological distress (Table II). The HADS-A results show that 25% of patients and 34% of NoK were possible or probable cases of anxiety. Moreover, according to the HADS-D, 14% of patients and NoK were possible or probable cases of depression.

**Table II:** Differences in self-reported well-being between patients and next of kin (NoK)

HADS summed score		Normal	Possible anxiety		Probable anxiety		P-value					

Anxiety	Pats N (%)	171 (75)	32 (14)		26 (11)		0.09					
	NoK N (%)	132 (66)	42 (21)		27 (13)							

		Normal	Possible depression		Probable depression		P-value					

Depression	Pats N (%)	196 (86)	21 (9)		12 (5)		0.92					
	NoK N (%)	172 (86)	17 (8)		12 (6)							

FACT-G items assessing well-being		Not at all (0)		A little bit (1)		Somewhat (2)		Quite a bit (3)		Very much (4)		P-value

		Pats N (%)	NoK N (%)	Pats N (%)	NoK N (%)	Pats N (%)	NoK N (%)	Pats N (%)	NoK N (%)	Pats N (%)	NoK N (%)	

GP1	I have a lack of energy	58 (26)	66 (33)	55 (24)	73 (37)	67 (29)	41 (21)	45 (20)	16 (8)	3 (1)	2 (1)	< 0.01
GP6	I feel ill	130 (58)	157 (80)	46 (20)	24 (12)	37 (17)	10 (5)	11 (5)	4 (2)	0 (0)	2 (1)	< 0.0001
GP7	I am forced to spend time in bed	177 (79)	183 (93)	29 (13)	5 (3)	12 (5)	6 (3)	5 (2)	3 (1)	1 (1)	0 (0)	0.02
GE1	I feel sad	53 (23)	54 (27)	76 (34)	65 (32)	64 (28)	53 (26)	28 (12)	26 (13)	6 (3)	3 (2)	0.53
GF1	I am able to work	10 (4)	4 (2)	22 (10)	3 (1)	58 (25)	14 (7)	97 (43)	70 (35)	40 (18)	110 (55)	< 0.0001
GF3	I am able to enjoy life	7 (3)	3 (2)	18 (8)	11 (5)	55 (24)	44 (22)	95 (42)	86 (43)	53 (23)	57 (28)	0.11
GF7	I am content with my QoL	18 (8)	9 (4)	22 (10)	18 (9)	58 (25)	43 (21)	86 (38)	85 (43)	44 (19)	45 (23)	0.11

FACIT-Sp-12 items assessing well-being		Not at all (0)		A little bit (1)		Somewhat (2)		Quite a bit (3)		Very much (4)		P-value

		Pats N (%)	NoK N (%)	Pats N (%)	NoK N (%)	Pats N (%)	NoK N (%)	Pats N (%)	NoK N (%)	Pats N (%)	NoK N (%)	

Spl	I feel peaceful	14(6)	10(5)	14(6)	13(6)	50(22)	53(27)	102(46)	88(44)	45 (20)	35 (18)	0.54
Sp2	I have a reason for living	0(0)	0(0)	1(0)	1(0)	6(3)	8(4)	36(16)	35(18)	184 (81)	156 (78)	0.58
Sp5	I feel a sense of purpose in my life	0(0)	0(0)	5(2)	0(0)	14(6)	15(8)	59(26)	48(24)	150 (66)	135 (68)	0.81
Sp8	My life lacks meaning and purpose	173(76)	146(73)	28(12)	19(10)	17(8)	17(8)	5(2)	5(2)	4 (2)	13 (7)	0.41

Data show the percentage of participant-reported answers to the HADS, and selected FACT-G and FACIT-Sp-12 items. χ^2^ test was used to analyse the differences in grouped HADS scores Mann-Whitney U test was used to analyse the differences in FACT-G and FACIT-Sp-12 items between groups.

GE = General Emotional; GF = General Functional; GP = General Physical; NoK = next of kin (N = 197-201); Pats = patients (N = 224-229); QoL = quality of life. A few participants did not answer all items, hence the variable N per item.

No significant differences in well-being between patients and NoK were found for most items (see Table II). The FACIT-Sp-12 results showed no differences between patients and NoK. Patients reported a higher impact on the FACT-G items lack of energy, feeling ill, and being forced to spend time in bed than NoK.

**Fig. 1:**
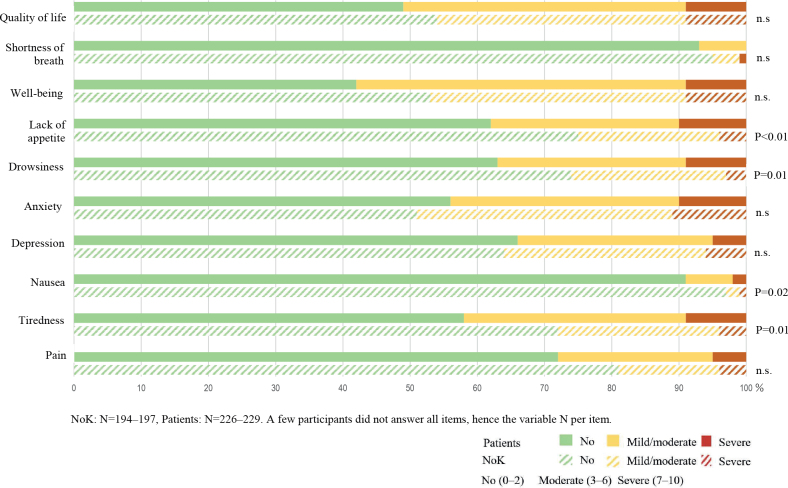
***χ*^2^ test results of grouped ESAS scores.** Differences between patients and next of kin (NoK) are shown. n.s.: not significant.

In addition to the HADS results, self-reported ESAS responses showed an even higher frequency of participants reporting anxiety and depression (Fig. 1). Almost half of NoK reported a mild/moderate to severe impact on anxiety, as did 44% of patients. More than one-third of patients and NoK reported a mild/moderate to severe impact on depression. Additionally, about half of the participants reported a mild/moderate to severe impact on the ESAS items well-being and QoL. In items targeting physical well-being and daily activities, e.g., the ESAS items appetite, drowsiness, nausea, and tiredness, the patients reported higher scores than the NoK (see Fig. 1).

### Unmet rehabilitation needs

In all 26 compared CaSUN/CaSPUN items, most participants reported no unmet need, or that their needs were being met. Yet in each domain there was a moderate prevalence (≈10–25%) of participants who did report unmet needs, most frequently in the information and psychosocial domains. Table III lists the 12 most frequently reported unmet rehabilitation needs (reported by at least 15% of patients or NoK) before start of adjuvant CT. Mean values (possible range 1–3) of the strength of the unmet need were between 1.4 and 2.2.

**Table III:** **The twelve most frequently reported unmet needs.** χ^2^ test was used to analyse differences in CaSUN/CaSPUN scores between patients and NoK.

CaSUN/CaSPUN need items	Domain	No need		Need being met		Unmet need		P-value
		Pats N (%)	NoK N (%)	Pats N (%)	NoK N (%)	Pats N (%)	NoK N (%)	
1 Up to date information	Information	9 (4)	15 (8)	174 (82)	142 (73)	30 (14)	38 (19)	0.16
2 My family and/or partner needs relevant information	Information	23 (11)	16 (8)	148 (69)	133 (68)	43 (20)	47 (24)	< 0.0001
3 Information provided in a understandable way	Information	23 (11)	34 (17)	156 (73)	121 (61)	34 (16)	42 (22)	0.11
7 To know that all doctors coordinate the care	Comprehensive care	55 (26)	73 (37)	131 (61)	75 (38)	29 (13)	50 (25)	< 0.0001
10 Help to reduce stress in my life	Psychosocial	162 (73)	133 (67)	35 (16)	31 (16)	24 (11)	33 (17)	0.39
11 Help to manage side effects and/or complications of treatment	QoL	126 (57)	125 (63)	81 (36)	44 (22)	15 (7)	28 (15)	0.01
15 Help to find out about financial support/goverment benefits	Other	170 (77)	128 (65)	29 (13)	23 (12)	22 (10)	45 (23)	< 0.01
19 Help to manage my concerns about cancer coming back	Psychosocial	142 (64)	128 (65)	34 (15)	20 (10)	47 (21)	49 (25)	0.13
20 Emotional support	Psychosocial	109 (50)	126 (63)	85 (39)	41 (21)	25 (11)	32 (16)	< 0.01
21 Help to know how to support my partner and/or family	Relationship	154 (70)	129 (64)	28 (13)	28 (14)	38 (17)	43 (22)	0.39
26 Help to adjust to body changes due to cancer	Psychosocial	153 (69)	174 (87)	35 (16)	10 (5)	34 (15)	16 (8)	< 0.01
28 Help and support with services when required	Other	139 (67)	137 (69)	47 (22)	31 (16)	23 (11)	30 (15)	0.03

NoK = next of kin (N=194–202); Pats = patients (N=209–225); QoL = quality of life. A few participants did not answer all items, hence the variable N per item.

One-fifth of patients and almost 25% of NoK reported an unmet need for information: “My family and/or partner needs relevant information”. More than a quarter of patients and almost one-fifth of NoK reported the psychosocial unmet need “I need help to manage my concerns about cancer coming back”. In the informational domain, ≈10–15% of participants reported “no need”, while ≈60–80% reported their “need being met”. In psychosocial, and relationship domains, results were the opposite, as 50–87% of participants reported “no need” and 5–40% answered “need being met”.

Significant group differences were seen in 12 of the 26 CaSUN/CaSPUN items. In 10 of these, the prevalence of unmet needs was greater among NoK than among patients. The largest frequency discrepancy was reported for “I need to know that all doctors coordinate the care” (25% of NoK, 13% of patients) and “I need help finding out about financial support/government benefits” (23% of NoK, 10% of patients).

## DISCUSSION

As far as we know, this is the first study to assess pre-CT well-being and unmet needs among cancer patients and their NoK in northern Sweden. Our findings highlight the impact of cancer on well-being, and the unmet needs experienced by both patients and NoK. The HADS results reveal that a moderate proportion of participants had increased psychological distress. The ESAS scores identified even more participants with possible psychological distress. In contrast, one-third of participants met our pre-set criterion for no/minimal impact on well-being.

For all CaSUN/CaSPUN items, most participants reported no unmet need, or their needs being met. Yet in each domain there was a moderate prevalence (≈10–25%) of participants who did report unmet needs, most frequently in the information and psychosocial domains.

### Well-being and psychological morbidity

Both anxiety and depression conditions are common psychological complications of cancer, but the reported prevalence among cancer patients varies greatly. Several factors can explain this variability, e.g., cancer diagnosis, stage of cancer, in- or outpatient care, and different assessment tools used (25). Sociocultural and economic factors as well as geographic inaccessibility to specialized services are contextual factors also suggested to impact the psychological outcomes for cancer patients (26, 27). Our findings of 14% of individuals with a possible or probable impact on depression is consistent with the in-depth meta-analysis by Mitchell et al. with data on 10,071 patients in oncological and haematological settings, diagnosed at early or mixed stages, which showed a prevalence of depression of ~16%. However, Mitchell et al. demonstrated a prevalence of anxiety of ~10%, which differs from our 25%. (28).

Latham et al. found in a systematic review (SR) that emotional/mental HRQoL in rural and urban cancer survivors was generally similar to that in normative populations (5). This is consistent with the findings in a large SR by Van der Kruk et al., who reported no differences in psychosocial morbidity and QoL between rural and urban cancer survivors in Australia and other developed countries (6). When well-being in patient–caregiver dyads was compared in a recent study by Goodwin et al. in regional/remote Australia, results suggested that NoK had a significantly higher QoL in the physical and mental QoL dimensions. Anxiety among NoK was significantly lower than patient anxiety, but NoK and patients reported similar levels of depression and stress (29). This partly differs from the findings presented in our study, where NoK and patients reported similar levels of both anxiety and depression (HADS and ESAS).

It is important to consider that time since diagnosis and treatment phase may affect the risk of psychological morbidity among cancer patients. Previous studies demonstrate a tendency for increased depression during the acute phase, which decreases after treatment. Our findings align with those of a meta-analysis by Krebber et al., which reported a pooled prevalence of depression of 14% during the acute phase (30).

Our work adds to the growing body of research regarding the psychological morbidity of cancer, by suggesting that a moderate proportion of both patients and NoK in the outpatient context of northern Sweden are affected by anxiety (25% and 34%, respectively) and depression (14%). This is important because there is reason to believe that anxiety and depression negatively affect the overall well-being of patients and NoK (31–33).

### Unmet rehabilitation needs

Consistent with Harrison et al., who reviewed 57 articles and identified the most frequently reported unmet needs in daily activities, psychological, informational, psychosocial, and physical domains among cancer patients, our study found most unmet needs within informational and psychosocial domains. In their SR, unmet needs were shown to be the highest and most varied during ongoing active cancer treatment. However, they also identified a greater number of patients expressing unmet needs post-treatment rather than at any other time (34). The longitudinal design of our study will generate further results and enable comparison over time. Lambert et al. reviewed the unmet needs of caregivers to cancer patients in 29 articles and found unmet needs in all domains. Half of the included studies reported that up to a third of caregivers had unmet needs in the emotional/psychological, partner/caregiver-related, daily activities, and informational domains, results being a bit higher than ours, where up to a quarter of NoK-reported unmet needs (35). A recent SR of 65 studies, by van der Kruk et al., found that rural cancer patients and their caregivers had specific unmet psychosocial needs related to accessing care, and to travel and finances. However, most studies reported no significant differences in psychosocial needs between rural and urban groups (6).

This study found a moderate prevalence (≈15–25%) of unmet needs in informational and psychosocial domains among both patients and NoK. These results domains among both patients and NoK. These results corroborate the findings of previous work mentioned earlier (34, 35); however, our results add to these by comparing patients and their NoK. We found no clinically significant differences in unmet needs between patients and NoK for most compared items, but, when a difference was apparent, NoK generally reported greater levels of unmet needs than patients. This underlines the importance of assessing unmet needs in clinical routine practice and not forgetting to include NoK, as the proportion of unmet rehabilitation needs among NoK tends to exceed that of patients.

### Limitations

We acknowledge several limitations in this study. First, the assessment of well-being is complex, in terms of both definition and the actual assessment, with no consensus or agreement. By focusing on the *objective functioning* aspects of well-being, specifically psychological distress (anxiety and depression), we have assessed an important part of well-being. The choice to include selected items from the FACT-G and FACIT-Sp-12 allowed for an easier assessment of aspects of physical and psychological well-being; however, the study does not claim to fully account for all aspects of the well-being concept.

The discrepancy between our results using the HADS and the ESAS in assessing the impact on anxiety and depression should be noted. The prevalence of possible and probable depression in patients was 14% according to the HADS and 34% according to the ESAS. Vodermaier and Millman’s meta-analysis, which pooled HADS results for cancer patients, indicated that the optimal thresholds were 5 for depression (HADS-D) and 7 or 8 for anxiety (HADS-A) (36). Elsewhere, ESAS scores with a cut-off ≥ 3 have been identified as effective in screening for anxiety and depression (37), while a meta-analysis by Boonyathee et al. suggested that an ESAS-D score ≥ 4 may indicate possible depression in patients with cancer (16). The chosen cut-offs in our study (HADS > 7 and ESAS ≥ 3) may have been somewhat strict for the HADS and a little too liberal for the ESAS.

We did not register comorbidity, but patients for whom CT was contraindicated were excluded from the study. Comorbidities can affect outcomes in complex ways, thus complicating the cancer rehabilitation process. Neither did we adjust for confounders, but the patients and NoK had similar sociodemographic characteristics and vulnerability factors at baseline.

Despite the extensive questionnaire package, some potentially important rehabilitation areas have not been covered. This study did not explore physical activity, support from a physiotherapist and/or dietitian, or participation in patient and family organizations, all important factors possibly impacting well-being and the development of unmet needs over time (38, 39).

### Clinical relevance and implications for further studies

The study participants were a mixed group of patients with different diagnoses and treatments (even though most patients had breast cancer and were female), and NoK with different relationships to the patient. This mix is representative of the typical adjuvant cancer patient population in northern Sweden, and hence has greater clinical relevance than a more limited population.

In a cancer care context where both financial funds and healthcare providers are scarce, a clinical approach to identify those with the largest and smallest need is desirable, which can also help identify areas to be prioritized for assessment. Our study identified one-third of participants reporting a negative psychological impact of cancer, as well as one-third with no/minimal impact, i.e., participants feeling relatively well. This finding is reassuring because it strengthens the feasibility of screening to find those individuals with the most pronounced needs. Furthermore, our findings demonstrate differences in well-being and unmet needs among participants, suggesting that a one-size-fits-all approach to cancer care and rehabilitation is inadequate.

In conclusion, our data suggest that NoK in several cases experience a similar impact on psychological distress to patients, and experience unmet needs at the same level as or a higher level than patients. This emphasizes the need to include NoK in an early rehabilitative approach. Taken together, individual assessment of well-being and unmet needs should be prioritized for both patients and NoK early in the cancer trajectory.
